# Supplementary material for: Seminoma and Embryonal Carcinoma Footprints Identified by Analysis of Integrated Genome-Wide Epigenetic and Expression Profiles of Germ Cell Cancer Cell Lines
Source: PLoS One. 2014 Jun 2;9(6):e98330. doi: 10.1371/journal.pone.0098330 (PMC4041891; doi:10.1371/journal.pone.0098330)
Supplement: File S1 — ZIP file containing DMRforPairs output for significant regions. Please start from the html files. (ZIP) [file pone.0098330.s008.zip › figures/14580.pdf]

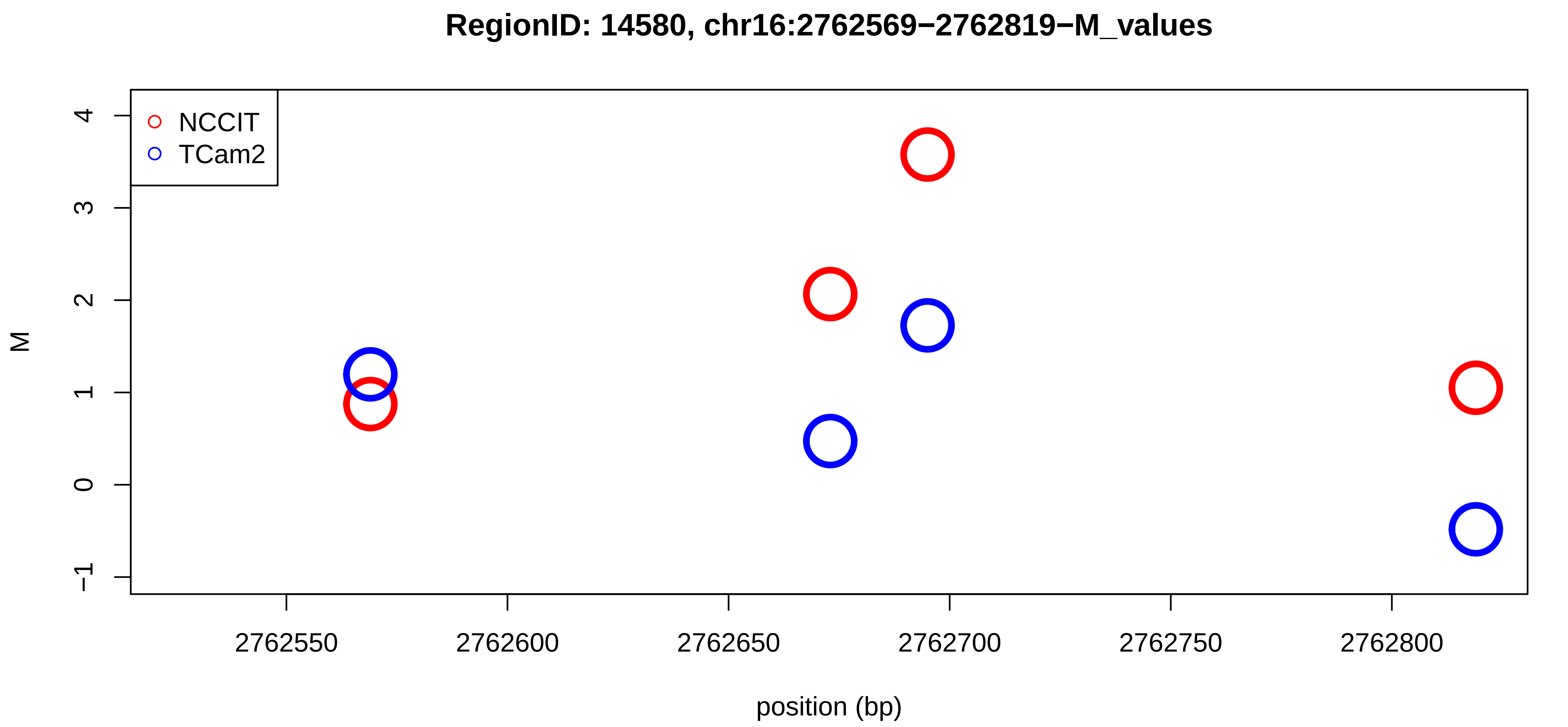

RegionID: 14580, chr16:2762569−2762819−M_values
NCCIT
TCam2
M
−1
0
1
2
3
4
2762550
2762600
2762650
2762700
2762750
2762800
position (bp)

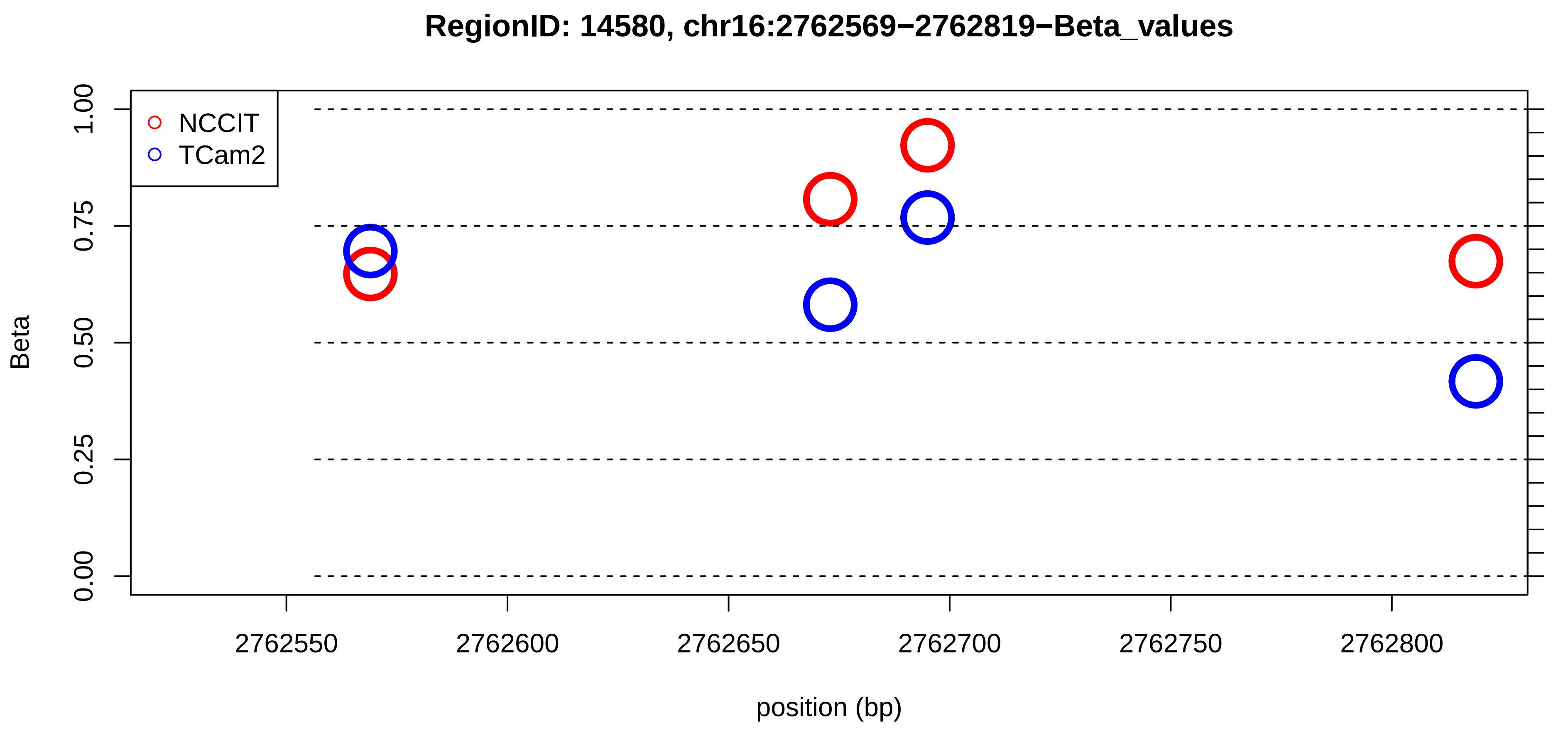

RegionID: 14580, chr16:2762569−2762819−Beta_values
NCCIT
TCam2
Beta
0.00
0.25
0.50
0.75
1.00
2762550
2762600
2762650
2762700
2762750
2762800
position (bp)
